# A comparison of three induction regimens using succinylcholine, vecuronium, or no muscle relaxant: impact on the intraoperative monitoring of the lateral spread response in hemifacial spasm surgery: study protocol for a randomised controlled trial

**DOI:** 10.1186/1745-6215-13-160

**Published:** 2012-09-08

**Authors:** Yuan Fang, Heng Zhang, Wenke Liu, Yu Li

**Affiliations:** 1 Department of Neurosurgery, West China Hospital, Sichuan University, 37 Guo Xue Xiang Street, Chengdu, 610041, China; 2 Department of Anesthesiology, West China Hospital, Sichuan University, 37 Guo Xue Xiang Street, Chengdu, 610041, China

**Keywords:** Hemifacial spasm, Microvascular decompression, Muscle relaxant, Succinylcholine, Vecuronium, Intraoperative monitoring, Lateral spread response

## Abstract

**Background:**

Surgical microvascular decompression (MVD) is the curative treatment for hemifacial spasm (HFS). Monitoring MVD by recording the lateral spread response (LSR) intraoperatively can predict a successful clinical outcome. However, the rate of the LSR varies between trials, and the reason for this variation is unclear. The aim of our trial is to evaluate the rate of the LSR after intubation following treatment with succinylcholine, vecuronium, or no muscle relaxant.

**Methods and design:**

This trial is a prospective randomised controlled trial of 96 patients with HFS (ASA status I or II) undergoing MVD under general anaesthesia. Patients are randomised to receive succinylcholine, vecuronium, or no muscle relaxant before intubation. Intraoperative LSR will be recorded until dural opening. The primary outcome of this study is the rate of the LSR, and the secondary outcomes are post-intubation pharyngolaryngeal symptoms, the rate of difficult intubations, the rate of adverse haemodynamic events and the relationship between the measurement of LSR or not, and clinical success rates at 30 days after surgery.

**Discussion:**

This study aims to evaluate the impact of muscle relaxants on the rate of the LSR, and the study may provide evidence supporting the use of muscle relaxants before intubation in patients with HFS undergoing MVD surgery.

**Trials registration:**

http://www.chictr.org/ ChiCTR-TRC-11001504 Date of registration: 24 June, 2011.

The date the first patient was randomised: 30 September, 2011.

## Background

Primary hemifacial spasm (HFS) is a disorder that causes frequent involuntary contractions in the muscles on one side of the face, due to a blood vessel compressing the nerve at its root exit zone (REZ) from the brainstem [1]. Numerous prospective and retrospective case series have confirmed the efficacy of microvascular decompression (MVD) of the facial nerve in patients with HFS with low rates of symptom recurrence and transient complications [1].

**Figure 1:**
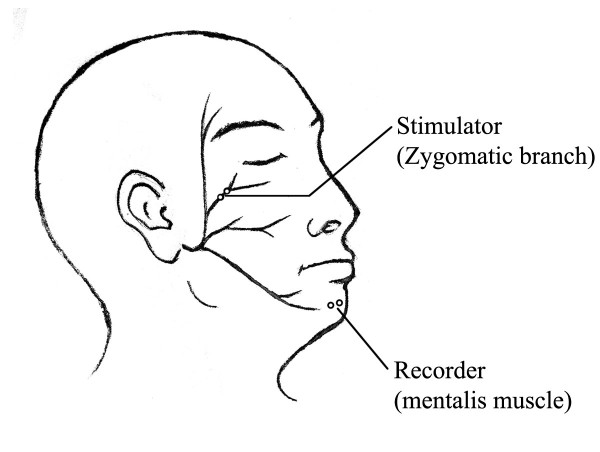
Schematic illustration shows the stimulation and recording electrodes.

In 1985, Møller and Jannetta [2] showed that in HFS, stimulation of one branch of the facial nerve activates facial muscles innervated by another branch, thereby producing abnormal muscle responses (AMR). These AMR are known as the lateral spread response (LSR) and can be recorded from one muscle innervated by the superior branch of the facial nerve when the inferior branch is stimulated or vice versa (Figure 1). Due to the fact that the LSR disappears instantly in most patients when the offending vessel is moved off the facial nerve, monitoring the AMR can guide the surgeon during MVD, which results in a better post-operative outcome [3]. Although the practical value of the LSR disappearance as a method to evaluate MVD efficacy is still controversial [4,5], in most cases, LSR monitoring is an effective tool to predict outcome after MVD for HFS [6].

However, an accurate estimate of the LSR rate is not yet known; the rate varies from 75% to 100% [7-14] between trials. Patients without the LSR would not benefit from this technology.

The reasons why the LSR is not detectable in some patients are unclear. Kong *et al*. hypothesised that the absence of the LSR was associated with the dosage of the muscle relaxant or the insertion site of the needle [11]; other researchers hypothesised that it was related to a process of denervation-reinnervation caused by preceding botulinum toxin injections [9]. However, these hypotheses have not yet been verified. While consulting the literature, we found that the muscle relaxants used in the various trials differed, which might be an important factor associated with the LSR rate. In most studies, short-acting muscle relaxants were used [1,7,9,10,13,15]. Sekula *et al*. [6] indicated that non-depolarising muscle relaxants should be used. While other studies described that muscle relaxants were used for intubation [4,14], they did not identify which muscle relaxants were used. Among the studies in the literature, several studies [11,13,16] performed the train of four (TOF) test to measure the degree of neuromuscular blockade and maintained the ratio at a level of 0.5 to 0.75.

### Succinylcholine

Succinylcholine (Sch) is an ultra-short-acting depolarising neuromuscular blocking agent (NMBA). This agent inhibits the action of acetylcholine at the neuromuscular junction. At a dose of 1 mg/kg, the onset time of Sch is 4 seconds; the time to 25% recovery is 10 minutes, and the time to 95% recovery is 12 to 15 minutes [17].

### Vecuronium

Vecuronium is an intermediate-acting non-depolarising neuromuscular blocking agent. This agent acts by competing for cholinergic receptors at the motor end-plate. At a dose of 0.1 mg/kg, the onset time of vecuronium is 2.3 minutes; the time to 25% recovery is 45 to 60 minutes, and the time to 95% recovery is 60 to 80 minutes [17].

The primary objective of this study is to evaluate the rate of LSR in HFS patients undergoing MVD under general anaesthesia with tracheal intubation, with succinylcholine, vecuronium, or no muscle relaxant. The secondary objectives are post-intubation pharyngolaryngeal symptoms, ease of intubation, haemodynamic responses and the relationship between the measurement of LSR or not, and clinical success.

## Methods

This is a randomised controlled study comparing the LSR rate in HFS patients undergoing MVD under general anaesthesia with tracheal intubation, and prior treatment with succinylcholine, vecuronium, or no muscle relaxant.

This study is a three-arm, randomised controlled trial. Participants fulfilling eligibility criteria were selected. Enrolled participants were randomly allocated to three parallel groups: the Succinylcholine, Vecuronium, or No Muscle Relaxant.

### Inclusion and exclusion criteria

Inclusion criteria: adult patients (American Society of Anesthesia (ASA) status I or II) (Table 1) [18] diagnosed with primary HFS and treated with MVD under general anaesthesia will be enrolled.

**Table 1:** ASA physical status classification system

**Classification**	**Physical status**
1	A normal healthy patient.
2	A patient with mild systemic disease.
3	A patient with severe systemic disease.
4	A patient with severe systemic disease that is a constant threat to life.
5	A moribund patient who is not expected to survive without the operation.
6	A patient declared brain-dead whose organs are being removed for donor purposes.

Exclusion criteria: patients with: HFS secondary to aneurysms, tumours, or cysts; factors predictive of a difficult intubation; a BMI above 30 kg/m^2^; a history of allergy to muscle relaxants. Patients reporting any preoperative sore throat or hoarseness at history taking will be excluded.

**Figure 2:**
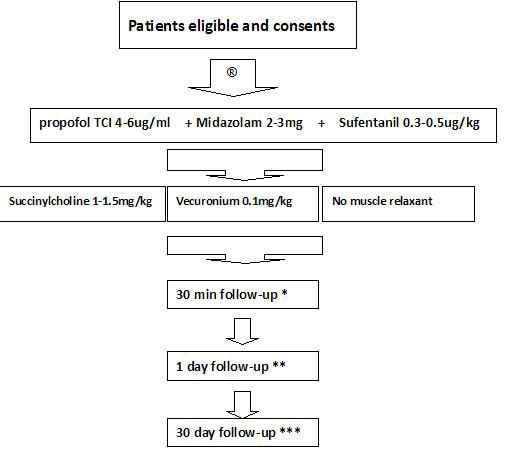
**Trial flow chart.** *Rate of the LSR. **Post-intubation pharyngolaryngeal symptoms, ease of intubation and haemodynamic responses. ***Post-surgical symptoms of hemifacial spasm.

All consenting patients fulfilling the inclusion criteria are randomised into three groups in a 1:1:1 ratio (G1, the Succinylcholine group; G2, the Vecuronium group; G3, the No Muscle Relaxant group). The flow chart of the present trial is shown in Figure 2.

Patient demographic data will be collected for age, sex, duration of symptoms, location, history of botulinum toxin injections prior to MVD, and offending vessel.

Blinding: double blinding is not possible because of the muscle fasciculation caused by succinylcholine [19]. Outcome assessors will be blinded.

### Randomisation

Block randomisation was performed using a computer-generated list containing a sequence of letters, G1 referring to the Succinylcholine group, G2 referring to the Vecuronium group and G3 referring to the No Muscle Relaxant group. Block sizes vary with three, six or nine letters. Allocations were concealed in opaque, sealed envelopes. The envelope contains a number that is concealed as to the allocation. The list will be generated and kept by a person not involved in the study in the operating room. The number of envelopes matches the number of patients ready for the randomisation and follows the sequential numbers on the generated list.

### Outcome measures

The primary outcome is the intraoperative LSR rate.

All patients undergo orotracheal intubation in the supine position with the head and neck in neutral positions. Standard clinical monitoring is performed. After preoxygenation through a face mask for 4 min, anaesthesia is induced with propofol (target-controlled infusion (TCI) 4 to 6 μg/ml), sufentanil 0.3 to 0.5 μg/kg, midazolam 2 to 3 mg, and either succinylcholine 1 to 1.5 mg/kg (G1), vecuronium 0.1 mg/kg (G2), or no muscle relaxant (G3). Tracheal intubation is attempted 90 s after the administration. Intubation is always performed by an experienced anaesthetist using endotracheal tubes (Lo-Contour; Mallinckrodt, Athlone, Ireland) with an internal diameter of 7.0 mm for female patients and 7.5 mm for male patients. Mechanical ventilation is controlled, with end-tidal carbon dioxide being maintained at 30 to 40 mm Hg [17]. Anaesthesia is maintained using total intravenous anaesthesia (TIVA) with propofol and sufentanil.

After intubation, a bipolar subdermal needle electrode is inserted subcutaneously over the zygomatic branches of the facial nerve on the side of HFS with a 0.5 to 1 cm spacing. Another bipolar needle electrode is placed in the mentalis muscles for LSR recordings. Electrical stimulation, consisting of square-wave pulses (intensity: 5 to 15 mA; duration: 0.2 ms) and electromyographic (EMG) recordings, are filtered through a 30 Hz to 3 kHz band pass (gain: 500 mV/division; analysis time: 50 ms) (Axon Epoch 2000 Systems, Hauppauge, NY, USA). To avoid nerve fatigue, the LSR is evoked with a 2 min interval until dural opening.

The LSR is defined as the EMG response recorded from the mentalis muscle by electrical stimulation of the zygomatic branch of the facial nerve, usually with a latency of approximately 10 ms.

The secondary outcomes for this study are post-intubation pharyngolaryngeal symptoms, ease of intubation, haemodynamic responses, clinical success rates and its relationship to the measurement of LSR or not at 30 days after surgery, as described below:

1. Post-intubation pharyngolaryngeal symptoms are defined as hoarse or sore throat 24 h after extubation. The severity of the complaint is assessed on a 101-point numerical rating scale (0 = no discomfort, 100 = extreme discomfort).

2. The rate of difficult intubations is defined as an IDS score >5. The IDS (Table 2) is the sum of the seven following variables: number of tracheal intubation attempts, number of operators who attempted intubation, number of alternative techniques used, glottic exposure (as defined by the Cormack and Lehane classification [20]), intensity of lifting force (normal or increased) applied during laryngoscopy, necessity for external laryngeal manipulation, and position of the vocal cords (Table 2) [21].

3. The rate of adverse haemodynamic events is defined as hypotension and bradycardia and the need to administer ephedrine or atropine.

4. Clinical success is defined as HFS relief at 30 days after surgery.

**Table 2:** IDS Score

**Parameter**	**Score**
Number of attempts > 1	N1
Number of operators > 1	N2
Number of alternative techniques	N3
Cormack grade	N4
Lifting force required	
Normal	N5 = 0
Increased	N5 = 1
Laryngeal pressure	
Not applied	N6 = 0
Applied	N6 = 1
Vocal cord mobility	
Abduction	N7 = 0
Adduction	N7 = 1

### Sample size calculation

We performed a pilot study of the LSR rate (following the same protocol as the proposed study) before writing the protocol. According to the results of our preliminary study, the Vecuronium group percentage is assumed to be 60%, the Succinylcholine group percentage is assumed to be 70%, and the No Muscle Relaxant group percentage is 100%. Effect size is assumed 0.4 corresponding to our preliminary result. A sample size of 27 patients for each study group with an allocation ratio of 1:1:1 for a total of 81 patients is required to achieve 90% power (alpha at 0.05) to detect a difference among the groups in the primary end-point variable (LSR rate) (G*Power 3.1.2 software). Allowing for a drop-out rate of 15% per group, we decided to recruit 96 participants.

### Statistical analysis

Double data entry will be done by assistants not participating in the study. Baseline characteristics will be described and compared for all three groups. All main analyses were based on the intention-to-treat patients.

The primary statistical analysis is performed at the end of the surgery. All three group comparisons will be conducted. LSR rates will be compared using the chi-square test and RR is assumed for the calculation. All the dichotomous (secondary) outcomes will be compared between the three treatment groups using the chi square (or Fisher’s exact) test. Continuous (secondary) outcome of the severity of the complaint will be compared using one-way ANOVA.

Statistical analyses will be carried out using the SPSS software (SPSS 18.0, Chicago, IL, USA). A *P* <0.05 will be considered statistically significant.

### Treatment discontinuation

If intubating conditions are unsatisfactory due to poor muscle relaxation, the second anaesthetist in attendance could administer succinylcholine (1 mg/kg) to patients in the No Muscle Relaxant group, but all patients randomized need to be included in the analyses.

### Ethical considerations

The study will be conducted according to the principles of the Declaration of Helsinki (2008 version) [22].

The Medical Ethics Committee of the West China Hospital, Sichuan University approved the protocol before start of the trial (6 Sep, 2011 Number 77; President Prof. Zeng Yong).

## Discussion

In the present study, we chose succinylcholine and vecuronium because they are the most frequently used neuromuscular blockers and are ultra-short-acting depolarising and intermediate-acting non-depolarising muscle relaxants, respectively. We did not choose rocuronium (the most rapid-onset non-depolarising relaxant) because it should be kept in the refrigerator at 2 to 8 degrees, which is unavailable in our operating room. We monitor the LSR from intubation to the dural opening, not during the whole surgery. Because the outflow of cerebrospinal fluid also causes a temporary shift in the neurovascular relationship equivalent to decompression, the LSR may disappear after the drainage of cerebrospinal fluid [7,9,13]. Anaesthesia is maintained using total intravenous anaesthesia (TIVA), because inhalation anaesthetics enhance the effect of muscle relaxants [23].

The mechanism of the LSR is controversial and may be a result of pulsatile compression at the REZ of the facial nerve [24]. The LSR has been related to cross-transmission (ephaptic transmission) of antidromic activity that occurs at a central site in the facial nucleus [25], but recently, Møller *et al*. has demonstrated that the abnormal responses are due to hyperactivity of the facial motor nucleus [26].

An important limitation that must be noted is that we do not routinely perform the TOF to measure the degree of the muscle relaxation. We plan to perform the TOF of the orbicularis oculi to study the relationship between the degree of muscle relaxation and the LSR in further studies.

To the best of our knowledge, this trial is the first attempt to investigate the LSR rate and the impact of muscle relaxants on the LSR. The results of this trial will have a major impact on the induction regimen of anaesthesia in patients with HFS undergoing MVD.

## Trial status

The trial was started in September 2011. To date, more than 30 patients have been included.

## Abbreviations

AMR: Abnormal muscle response; ASA: American Society of Anesthesia; EMG: Electromyography; HFS: Hemifacial spasm; LSR: Lateral spread response; MVD: Microvascular decompression; NMBA: Neuromuscular blocking agent; REZ: Root exit zone; Sch: Succinylcholine; TCI: Target-controlled infusion; TIVA: Total intravenous anaesthesia; TOF: Train of four.

## Competing interests

The authors declare no financial or competing interests.

## Authors’ contributions

YF and HZ conceived and designed the study, drafted the manuscript, and contributed equally to this work. WL contributed to the design of the study. YL conceived and designed the study and revised the manuscript. All authors read and approved the final manuscript.
